# Exploring depth‐related patterns of sponge diversity and abundance in marginal reefs

**DOI:** 10.1002/ece3.11643

**Published:** 2024-07-02

**Authors:** Juliano Morais, Igor L. Cordeiro, Aline P. M. Medeiros, George G. Santos, Bráulio A. Santos

**Affiliations:** ^1^ College of Science and Engineering James Cook University Townsville Queensland Australia; ^2^ Universidade Federal da Paraíba, Centro de Ciências Exatas e da Natureza, Departamento de Sistemática e Ecologia Cidade Universitária João Pessoa Paraíba Brazil; ^3^ Instituto de Formação de Educadores Universidade Federal Do Cariri Brejo Santo Ceará Brazil; ^4^ Programa de Pós‐Graduação Em Diversidade Biológica e Recursos Naturais (PPGDR), Centro de Biológicas e da Saúde (CCBS), Pimenta Universidade Regional Do Cariri (URCA) Crato Ceará Brazil

**Keywords:** beta diversity, depth gradient, Hill numbers, marginal reef, mesophotic habitat, porifera

## Abstract

Marine sponges play a vital role in the reef's benthic community; however, understanding how their diversity and abundance vary with depth is a major challenge, especially on marginal reefs in areas deeper than 30 m. To help bridge this gap, we used underwater videos at 24 locations between 2‐ and 62‐meter depths on a marginal reef system in the Southwestern Atlantic to investigate the effect of depth on the sponge metacommunity. Specifically, we quantified the abundance, density, and taxonomic composition of sponge communities, and decomposed their gamma (*γ*) diversity into alpha (*α*) and beta (*β*) components. We also assessed whether beta diversity was driven by species replacement (turnover) or by nesting of local communities (nestedness). We identified 2020 marine sponge individuals, which belong to 36 species and 24 genera. As expected, deep areas (i.e., those greater than 30 m) presented greater sponge abundance and more than eightfold the number of sponges per square meter compared to shallow areas. About 50% of the species that occurred in shallow areas (<30 m) also occurred in deep areas. Contrarily to expectations, alpha diversity of rare (^0^ *D*_α_), typical (^1^ *D*_α_), or dominant (^2^ *D*_α_) species did not vary with depth, but the shallow areas had greater beta diversity than the deep ones, especially for typical (^1^ *D*_β_) and dominant (^2^ *D*_β_) species. Between 92.7% and 95.7% of the beta diversity was given by species turnover both inside and between shallow and deep areas. Our results support previous studies that found greater sponge abundance and density in deep areas and reveal that species sorting is stronger at smaller depths, generating more beta diversity across local communities in shallow than deep areas. Because turnover is the major driver at any depth, the entire depth gradient should be considered in management and conservation strategies.

## Graphical abstract

In this study, we investigated sponge diversity and abundance across a depth gradient in Southwestern Atlantic marginal reefs, revealing that while sponge abundance and density increase with depth, alpha diversity remains stable. Beta diversity, however, is higher in shallower areas due to environmental heterogeneity, predominantly driven by species turnover. These findings underline the importance of considering the entire depth gradient in conservation and management strategies for marine sponges.

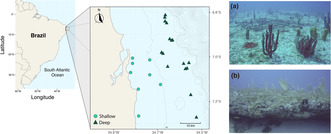

## 1 INTRODUCTION

Coral reefs provide habitat complexity that sustains the abundance, diversity, and ecosystem functioning of multiple reef‐associated communities (Coker et al., 2014; Darling et al., 2017). However, given the increasing impacts of climate change, widespread concerns have arisen about the abrupt loss of three‐dimensional reef structure caused by the mortality and rapid pos‐mortality erosion of reef‐building corals (Cornwall et al., 2021; Morais et al., 2022; Sully et al., 2019). In addition to global threats, such as climate change, local environmental conditions may also be unfavorable to reef‐build and reef‐associated communities. Although the urgent need for conserving and understanding the highly diverse typical coral reefs is unquestionable, it has been increasingly recognized that the research agenda of reef communities should also focus on less traditional reefs (Soares et al., 2021).

Known as “marginal reefs”, these formations occur under marginal or suboptimal conditions (Sommer, 2022). Despite being the focus of recent studies (Bleuel et al., 2021; Browne & Bauman, 2023; Morais & Santos, 2022; Sommer, 2022), these marginal habitats are still poorly explored, especially in the Southwestern Atlantic (Morais et al., 2018; Soares, 2020; Sommer, 2022). Marginal reefs are located in turbid‐zone, high‐sedimentation, high‐temperature regions, often associated with deeper zones, great nutrient concentration, high primary production rates, and extreme pH fluctuations, which can limit the distribution and occurrence of many reef species (Sexton et al., 2009; Soares et al., 2021). Thus, with the increasing impact of climate change, other benthic organisms, like sponges, can partially play an important role as reef builders and complexity structure providers (Bell, 2008; Buhl‐Mortensen et al., 2010; Diaz & Rützler, 2001; Wood, 1990) (but see Lesser & Slattery, 2020), especially where massive or upright sponges' growth forms are more common (e.g. the Tropical West Atlantic) (Bell et al., 2020). Given the importance of sponges as a potential provider of structural complexity in the absence of reef‐building corals, especially in marginal reef habitats under climate‐induced impact, studies focusing on the diversity and abundance of this group along depth gradients can broaden our understanding of marginal, deep turbid‐reefs (Moura et al., 2016), and subside effective management and conservation actions (Lesser, 2006).

Marine sponges are sessile and filter‐feeding animals (Vacelet & Boury‐Esnault, 1995) considered the oldest living metazoans, appearing around 800–900 million years ago (Muller, 2003). Beyond contributing to large biomass and playing a fundamental role in the structure of the benthic community (Fang et al., 2013; González‐Murcia et al., 2023), sponges may emerge as potential beneficiaries in the face of escalating global climate change threats (Bell, 2008). They play an important role as a source of food for a diverse array of marine species and participate in the nutrient cycling of dissolved organic matter (de Goeij et al., 2013). In addition to a range of other ecosystem services, sponges significantly enhance habitat diversity and structural complexity (Folkers & Rombouts, 2020). Different marine organisms use and depend on the sponge' structure at least in some stage of their life cycle (Bertelsen et al., 2009; Coppock et al., 2022). Furthermore, sponges are recognized as effective bioindicators owing to their stationary nature and filter‐feeding habits, which enable them to accumulate contaminants and participate in nutrient cycling (Folkers & Rombouts, 2020). The pharmacological industry also shows considerable interest in these organisms due to their ability to synthesize a vast array of bioactive compounds with antimicrobial, antiviral, antifungal, cytotoxic, and anti‐inflammatory properties (Fenical, 1996; Sipkema et al., 2005; Thakur & Müller, 2004).

Sponges and other benthic organisms respond to abiotic factors such as slope, coastal distance, exposure to wave energy, light incidence, temperature, pressure, sedimentation rate, and substrate composition (Moura et al., 2016; Sherman et al., 2010). Most of these factors are encapsulated into depth, resulting in directional conditional changes that affect marine communities along depth gradients (McArthur et al., 2010; Scott & Pawlik, 2018; Sexton et al., 2009). For example, Lesser (2006) and Lesser and Slattery (2018) demonstrated that sponges in the Caribbean benefit from greater nutrient availability (e.g., picoplankton; Ribes et al., 2003) in deeper reefs due to trophic interactions, which play a crucial role in shaping the ecosystem's structure. Consequently, this leads to a notable rise in both the abundance and biomass of sponges as the depth increases, indicating a direct correlation between the available nutrients in the deeper zones and the success of sponge populations therein (Reed & Pomponi, 1997).

Although the relationship between deeper areas and higher sponge abundance seems to be well explored in the literature (Scott & Pawlik, 2018), questions about the diversity and compositional differences between shallow and deep areas are still open. For example, we know little about how diversity is sorted across depth gradients: whether the regional (gamma) diversity is given by the sum of many species‐poor, but highly distinct local communities (high beta and low alpha diversity scenario), or by the replication of similar species‐rich local communities irrespective to depth (high alpha and low beta diversity scenario). The first scenario suggests that the metacommunity is structured by species sorting across particular environmental conditions, which differentiates local communities across space, while the second suggests that mass effects in favorable areas lead to the exportation of individuals toward less suitable zones, homogenizing the local communities across the gradient (Futuyma & Moreno, 1988; Jost, 2007; Leibold et al., 2004). It is also unknown whether beta diversity is generated by a high rate of species turnover between local communities, or because poorer local communities are subsets of a few richer communities (i.e. are nested within the richer communities) (Baselga, 2010).

Marginal reefs in Northeast Brazil (Southwestern Atlantic Province) rank among the South Atlantic's most biodiverse ecosystems (Leão et al., 2016; Spalding et al., 2007). At present, this region is recognized for harboring the most diverse sponge communities in Brazil, boasting approximately 290 documented species, followed by the Southeast region, with around 200 species (Santos, 2016). Despite the high diversity, the vertical and horizontal distributions of the sponge communities are still unexplored. Furthermore, although sponges occurring in shallow habitats (<20 m depth) were studied by Santos (2016) in this region, there is a lack of basic information regarding the abundance, density, and taxonomic composition in deeper areas (Feitoza et al., 2005). Similarly, there is no information on how regional diversity is influenced by species sorting and mass effects, and how turnover and nestedness underlie the diversity patterns. Describing these patterns can help us to disentangle the main drivers of metacommunity structure, as well as to draw effective management and conservation strategies (Medeiros et al., 2021; Pereira, Lima, Araujo, et al., 2022; Socolar et al., 2016).

In this study, we used video transects to investigate how sponge abundance, density, taxonomic composition, and diversity vary with depth across 24 southern Atlantic marginal reefs. Following the literature (Scott & Pawlik, 2018), we expected that sponge abundance (i.e. total number of individual sponges in each location) and density (i.e. number of individual sponges per square meter) were greater in deeper areas owing to higher resource availability at higher depths. The increase in depth should also lead to taxonomic differentiation between shallow and deep areas, as species should be sorted according to the environmental conditions imposed by depth. Alpha diversity was expected to increase with depth because food availability for filter‐feeding organisms is usually greater in deep areas (Lesser & Slattery, 2013). Beta diversity was expected to decrease with depth because the shallow locations are physically and biologically more heterogeneous to each other than the deep locations (Morais & Santos, 2018), generating more beta diversity in shallow areas. For this same reason, turnover should be the most important driver of beta diversity in shallow areas, while nestedness should underlie the beta diversity of deep areas.

## 2 METHODS

### 2.1 Study area

**FIGURE 1:**
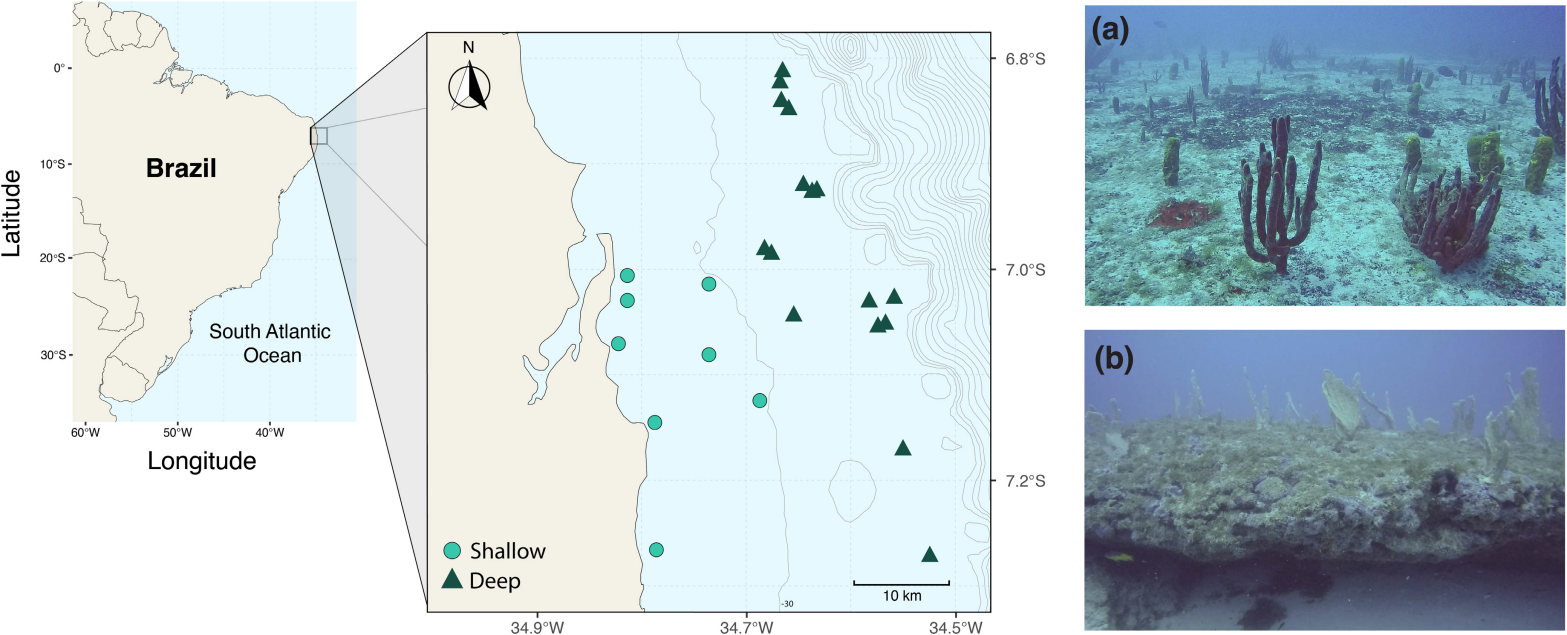
Map showing the sampling points in Paraiba State – Northeast Brazil (Southwestern Atlantic). Light green circles represent shallow reefs (<30 m), while dark green triangles represent deep reefs (>30 m). (a) Example of tubular and arborescent sponges established on a sand substrate. (b) Example of sponges established on reef substrate. Photographs by Ismar Dust and Orione Álvares.

The study was conducted in the continental platform of the southwestern Atlantic reef ecosystems located along the Northeastern Brazilian subprovince (Spalding et al., 2007), on the Paraiba State (Figure 1). This region is characterized by reef formations parallel to the coast, with isolated reefs varying in shapes and forms, and associated with sedimentary rocks (Leão & Dominguez, 2000). In the deeper areas, where the slope increases approaching the continental platform break, about 75 m depth, algae and sponges dominate the substrate (Feitoza et al., 2005). Water temperature varies between 28°C and 29°C, although a thermocline around 50 m depth decreases the temperature to 23°C and 24°C, gradually decreasing with depth (Feitoza et al., 2005).

### 2.2 Data survey

To investigate how the sponge community responds to depth, between January and February of 2017, we randomly sampled 24 areas between 2‐ and 62‐meter depth, in which 8 areas were considered shallow reefs (<30 m depth) and 16 areas were considered deep areas (>30 m depth). To sample sponge communities at each location, we performed SCUBA dives using compressed air, nitrox, or trimix depending on the depth. We then recorded four 20‐meter transects per location, accounting for a 1‐meter width on either side of the transect's centerline, yielding a total area of 40 square meters (20 × 2m), using high‐resolution GoPro cameras. Videos from different areas had different recording times, and the sampling was standardized by sample coverage rather than time, as recommended by Chao and Jost (2012). Specimens were meticulously identified from video records by the preeminent specialists in sponge taxonomy on our team, guided by the seminal “Systema Porifera” (Hooper et al., 2002) and incorporating the insights from more recent revisions such as Morrow and Cárdenas (2015). The distribution status of the identified sponges was based on the World Porifera Database (WPD) (de Voogd et al., 2024). It is critical to acknowledge that identifying sponge species solely through photo and video methodologies carries certain limitations compared to genetic analysis. Despite these constraints, our confidence in the identifications is bolstered by the expertise of the distinguished sponge taxonomist on our team. Moreover, there is a robust body of literature supporting species‐level identification via similar photo methodology (Carneiro et al., 2022; Moura et al., 2016; Pereira, Lima, Araujo, et al., 2022).

### 2.3 Data analysis

Sample coverage was measured in R (R Core Team, 2020), using the package *iNext* (Hsieh et al., 2016) and following the equation (Chao & Jost, 2012):

(1) $$\;{\hat{C}}_{n}=1-\frac{f_{1}}{n}\left[\frac{\left(n-1\right)f_{1}}{\left(n-1\right)f_{1}+2f_{2}}\right]$$

where *f*_1_ indicates the number of species with only one occurrence (i.e. singletons), *f*_2_ indicates the number of species with two occurrences (i.e. doubletons), and *n* is the overall number of individuals in each reef. Sample coverage was satisfactory in all reefs analyzed, with an average of 0.94, and varying between 0.64 and 1.00, which guarantees sampling efforts, besides indicating that the results are not biased by sampling.

To assess how depth affects species composition, we applied a non‐metric multidimensional scaling based on the Bray–Curtis similarity index (Clarke, 1993). Then, we performed a similarity analysis (ANOSIM) to compare species composition between shallow and deep regions. To test if depth affected the number of individual sponges per square meter (density), we used a generalized linear mixed effects model (GLMM) with a Gamma distribution with a log link function, and location as random intercepts in the model to account for any lack of spatial independence in the data. Model fit and assumptions were assessed using residual plots, all of which were satisfactory. To test if general sponge abundance (i.e. without considering surveyed area) and alpha diversity were positively related to depth categories, we performed a linear regression analysis (LM). Statistical modeling was performed in the software R (Team, 2020), using the “glmmTMB” package. (Brooks et al., 2017) and base “stats” package. As approached in other recent studies (Cardoso et al., 2020; Medeiros et al., 2021, 2022; Morais & Santos, 2018), beta diversity was compared with its absolute values plotted in the diversity profiles. The relative contribution of beta turnover and nestedness components were also compared between shallow and deep regions using absolute values.

Richness and abundance are commonly used to contrast diverse communities (Magurran, 2004). However, diversity index approaches better estimate biological variability in communities over time (Hill, 1973). While richness and abundance are related to simple counts of individuals, the term “diversity” offers a measure that encompasses both species number (i.e. richness) and evenness (i.e. equitability – how evenly the individuals in a community are distributed among the different species; Jost, 2006) (Gotelli & Chao, 2013; Hurlbert, 1971; Naeem et al., 2012). Most studies measure “diversity” by combining abundance and richness, which, although traditionally popular, are essentially measures of uncertainty or entropy and come with mathematical and biological constraints (Jost, 2006). To make an analogy, while these indices can give us a measurement similar to the radius of a sphere—which can tell us something about the sphere's size—they do not actually give us the sphere's volume. Just as relying solely on the radius to understand the full volume can lead to errors in fields like engineering, using these entropy‐focused diversity measures may not provide a complete picture of a community's diversity, potentially leading to misinterpretations when assessing ecological data (Jost, 2006). Entropy‐based metrics gauge the uncertainty of species identification within a sample, rather than the actual count of different species present, hence they often fall short of adhering to the “replication principle,” making comparisons across communities less realistic. Because of this, here we used Hill numbers, which is a family of diversity measures developed by Hill (1973). This metric quantifies diversity in units of equivalent numbers of equally abundant species (Gotelli & Chao, 2013), allowing to exponentially weight species abundance by a *q* factor and, unlike traditional diversity metrics, it satisfies the mathematical replication principle (see Chao et al., 2014; Jost, 2010). Hill's equation and its derivations (Chao et al., 2014; Hill, 1973; Jost, 2006), often called *true diversity*, are expressed by the effective number of species. ^0^ *D*, ^1^ *D*, and ^2^ *D* may be interpreted as the diversity of rare, typical, and dominant species. Moreover, these equations have the flexibility to be broken down into distinct alpha and beta components (Jost et al., 2010), a feature that renders them particularly suited for exploring the diversity of various marine organisms, such as sponges. This methodological flexibility allows for a nuanced understanding of diversity by separating the components that contribute to the overall diversity within a particular environment.

Building on this framework, we calculated alpha, beta, and gamma diversity using estimators founded on the effective number of species, commonly referred to as Hill numbers (Hill, 1973), in which abundances are weighted by a *q* factor (Jost, 2007). This allowed us to estimate the diversity of rare (^0^ *D*), typical (^1^ *D*), and dominant (^2^ *D*) species. Furthermore, these estimators satisfy the mathematical principle of replication (Gotelli & Chao, 2013), allowing a reliable comparison between communities with different sizes (i.e. different richness and abundance).

Gamma diversity (^*q*^ *D*_*γ*_) was calculated following the equation (Jost, 2007):

(2) Dγq=∑i=1Spiq1/1−q

where *S* represents the number of sponge species in the region, *p*_*i*_ represents the relative abundance of species *i*, and *q* represents the exponential parameter that determines the equation sensibility to species' relative abundance.

The average alpha diversity (^*q*^ *D*_*α*_) was based on the decomposition of gamma diversity as follows:

(3) Dαq=1N∑i=1Spi1q+1N∑i=1Spi2q+…1/1−q

where *p*_*i*_ is the relative abundance of the *i*‐th species in each one of the *N* local communities (i.e. each of the 24 reefs sampled (Jost, 2007).

To evaluate the patterns of beta diversity between shallow and deep regions, we used the multiplicative approach of diversity partition (Jost, 2007): ^*q*^ *D*_β_ = ^*q*^ *D*_*γ*_/^*q*^ *D*_*α*_. In this case, beta is given as the effective number of completely distinct communities, varying from 1, when all communities are identical, to *N*, when all *N* communities are completely distinct (Jost, 2007). Because we have 8 shallow reefs and 16 deep reefs samples, beta diversity can vary from 1 to 8 in shallow reefs, and from 1 to 16 in their deep counterparts. Alpha, beta, and gamma diversity were estimated in R using the package *entropart* (Marcon & Hérault, 2015).

To identify the potential drivers of beta diversity, we decomposed beta diversity in its turnover and nestedness components using the R package *betapart* (Baselga et al., 2020). For this purpose, we built a presence‐absence matrix and calculated beta diversity based on Jaccard's multi‐site dissimilarity index *β*_JAC_, which is a linear transformation of ^0^ *D*_β_ (see Arce‐Peña et al., 2021). Following this step, we partitioned *β*_JAC_ in its *β*_JTU_ (turnover) e *β*_JNE_ (nestedness) components for shallow and deep regions. *β*_JTU_ and *β*_JNE_ values are expressed in percentage of *β*_JAC_.

## 3 RESULTS

**FIGURE 2:**
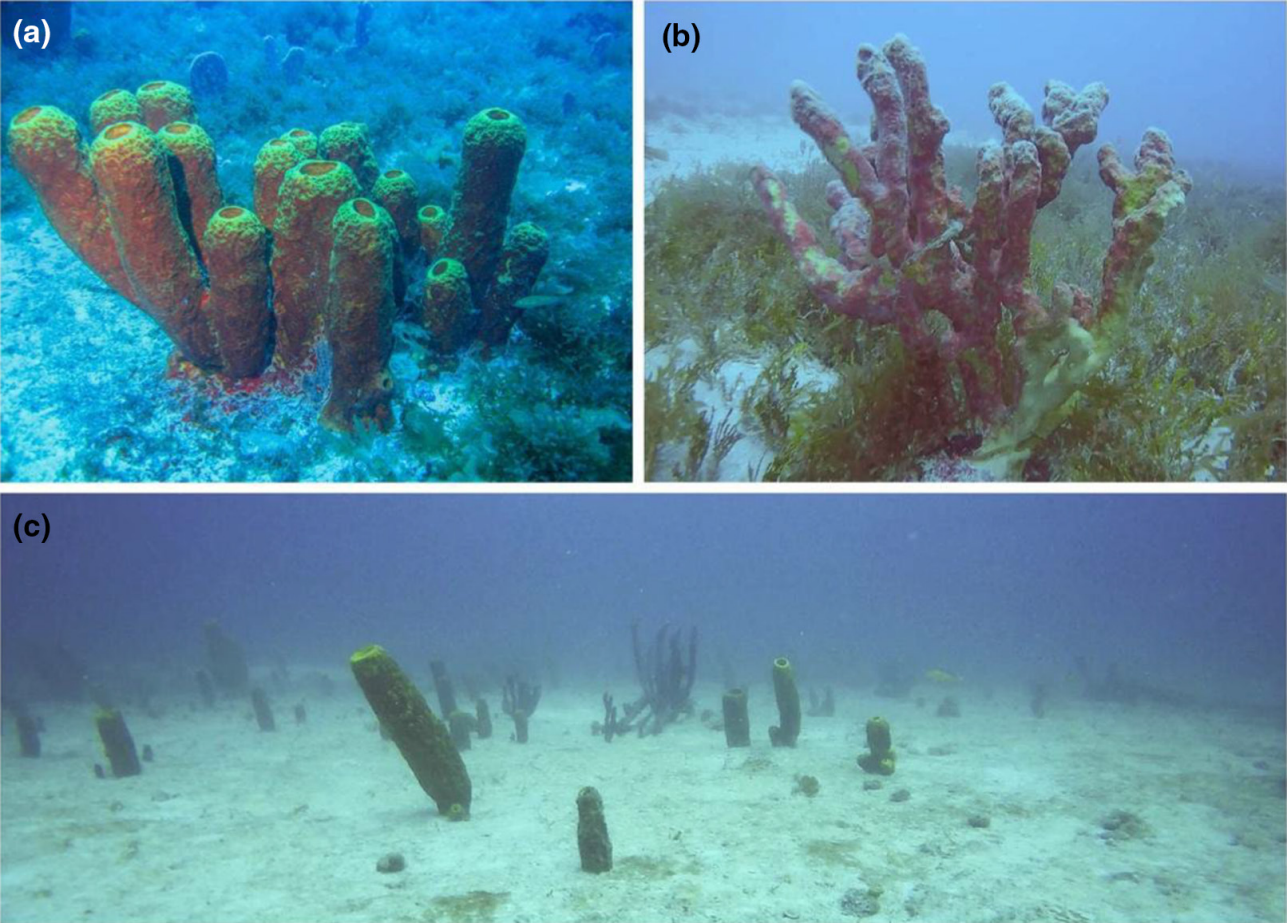
Examples of sponges in our sampling area. (a) a tubular sponge (*Aplysina lacunosa*) occurred in one of the deep habitats (>30 m). (b) an arborescent sponge (*Aplysina fulva*) in one of the shallow habitats (<30 m). (c) sponge community below 30 m depths in one of our study locations. Photographs by Ismar Dust and Orione Álvares.

Our survey revealed a total of 2020 marine sponge individuals across the depth gradient (i.e. 2–62 m). Within this count, shallow areas (<30 m) were home to 109 individual sponges, whereas the deep areas (>30 m) contained a significantly larger population of 1911 individuals. Notably, there was a high diversity within these communities, spanning 24 distinct genera and 36 species, showcasing the wide range of sponge life in the studied locations. Four of these species set new records for the region (i.e. Paraíba State — Brazil): *Agelas conifera* (Schmidt, 1870), *Siphonodictyon coralliphagum* (Rutzler, 1971), *Thorecta atlanticus* (Santos, et al, 2010) and *Xestospongia muta* (Schmidt, 1870). *Agelas conifera* and *X. muta* were recorded only in the deep region, while *T. atlantica* and *S. coralliphagum* occurred along all the depth gradient. Most abundant species were *Clathria* (*Clathria*) *nicoleae* (Barros et al. 2013) (27% of records), *Aplysina lacunosa* (Lamarck, 1814) (20.8%) and *Tedania (Tedania) ignis* (Duchassaing & Michelotti, 1864) (18%). Thirteen species occurred along the depth gradient (shallow and deep regions), while 7 were registered only in the shallow and 16 only in the deep region (Table 1). In deeper areas, the most dominant species were those tubular forms with great size, which provide more structural complexity to the system, such as *A. lacunosa* (Figure 2a,c), *C*. (*C*.) *nicoleae*, *T. atlantica* and *T. (T.) ignis*, while in the shallow areas most were encrusting species (Table 1).

**TABLE 1:** Sponge species recorded along the depth gradient in the continentals platform of Paraíba State, Northeast Brazil.

Species	Shallow reefs (abundance)	Shallow reefs (density ind/m^2^)	Deep reefs (abundance)	Deep reefs (density ind/m^2^)
*Agelas clathrodes* (Schmidt, 1870)			2	0.0008
*Agelas conifera* (Schmidt, 1870)			1	0.0004
*Agelas schmidtii* (Wilson, 1902)			1	0.0004
*Agelas* sp.			1	0.0004
*Aiolochroia crassa* (Hyatt, 1875)	4	0.0031		
*Amphimedon compressa* Duchassaing & Michelotti, 1864			8	0.0031
*Amphimedon viridis* Duchassaing & Michelotti, 1864			2	0.0008
*Aplysina fulva* (Pallas, 1766)	2	0.0016	179	0.0699
*Aplysina lacunosa* (Lamarck, 1814)	7	0.0055	414	0.1617
*Aplysina* sp.		0.0000	1	0.0004
*Biemna* sp.	1	0.0008		
*Callyspongia* sp.			1	0.0004
*Chondrilla caribensis* Rützler, Duran & Piantoni, 2007			2	0.0008
*Cinachyrella alloclada* (Uliczka, 1929)			9	0.0035
*Cladocroce caelum* Santos, Da Silva, Alliz & Pinheiro, 2014			45	0.0176
*Clathria* (*C*.) *nicoleae* Barros, Santos & Pinheiro, 2013	18	0.0141	527	0.2059
*Clathria* sp.	1	0.0008		
*Clathria* (*Thalysias*) *venosa* (Alcolado, 1984)	5	0.0039	4	0.0016
*Cliona varians* (Duchassaing & Michelotti, 1864)			32	0.0125
*Dragmacidon reticulatum* (Ridley & Dendy, 1886)			8	0.0031
*Dysidea etheria* de Laubenfels, 1936	2	0.0016	9	0.0035
*Echinodictyum dendroides* Hechtel, 1983	4	0.0031		
*Halichondria* (*H*.) *marianae* Santos, Nascimento & Pinheiro, 2018	3	0.0023	16	0.0063
*Haliclona* (*Reniera*) sp.	3	0.0023		0.0000
*Haliclona* (*Reniera*) *implexiformis* (Hechtel, 1965)	3	0.0023		
*Ircinia felix* (Duchassaing & Michelotti, 1864)	4	0.0031	13	0.0051
*Ircinia* sp.	12	0.0094		
*Ircinia strobilina* (Lamarck, 1816)	2	0.0016	137	0.0535
*Monanchora arbuscula* (Duchassaing & Michelotti, 1864)	3	0.0023	6	0.0023
*Niphatidae* sp.			1	0.0004
*Petrosiidae* sp.			2	0.0008
*Placospongia* sp.	3	0.0023	1	0.0004
*Siphonodictyon coralliphagum* Rutzler, 1971	9	0.0070	22	0.0086
*Tedania* (*Tedania*) *ignis* (Duchassaing & Michelotti, 1864)	14	0.0109	350	0.1367
*Thorecta atlanticus* Santos, Da Silva, Bonifácio, Esteves, Pinheiro & Muricy, 2010	9	0.0070	108	0.0422
*Xestospongia muta* (Schmidt, 1870)		0.0000	9	0.0035
Overall	109	0.0852	1911	0.7465

*Note*: Number in the shallow (<30 m depth) and deep (>30 m depth) reef categories represent species abundance and density (i.e. number of individuals per square meter).

**FIGURE 3:**
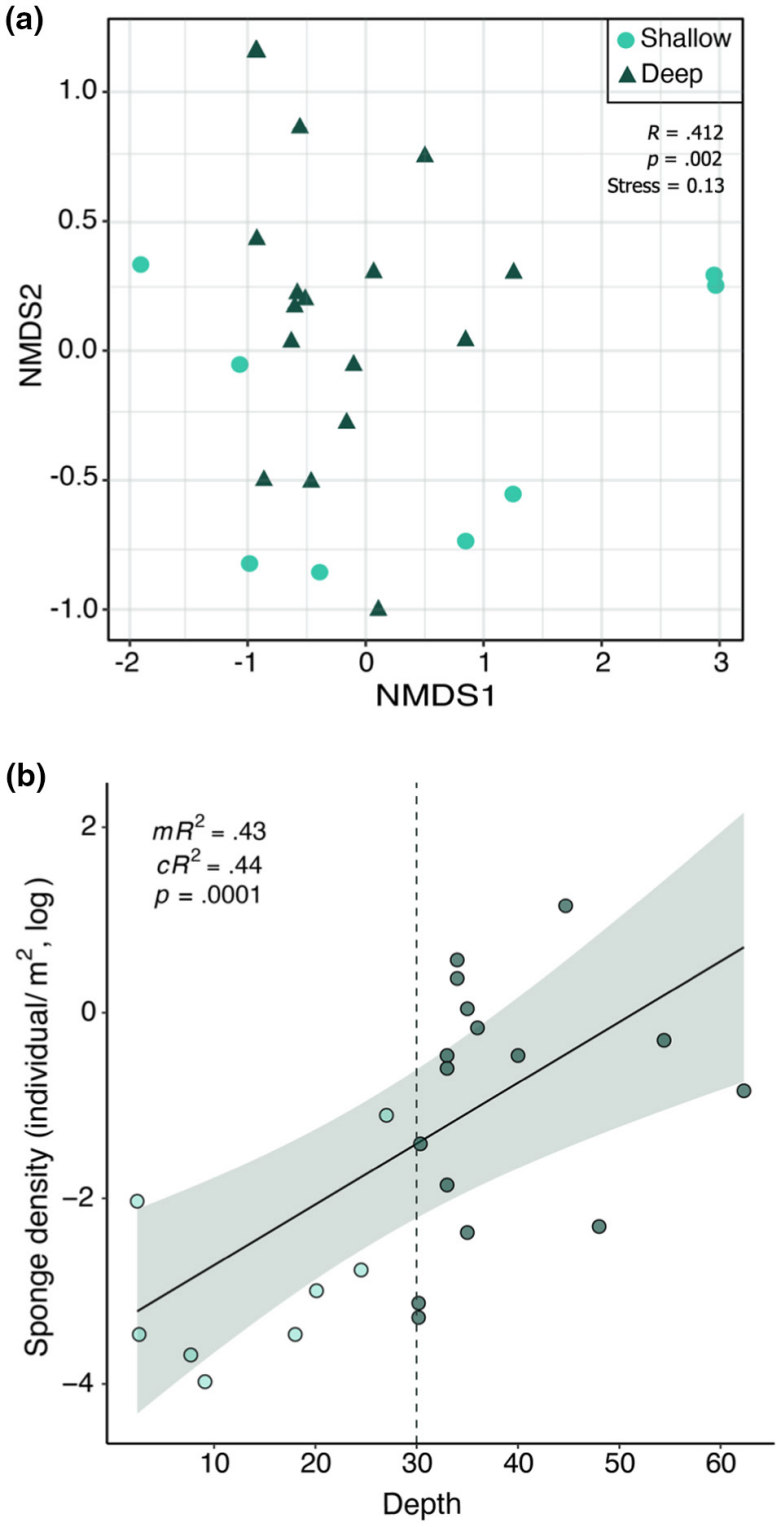
(a) Non‐multidimensional scaling for the 24 sponge communities analyzed in shallow (<30 m depth) and deep (>30 m depth) reefs along the continental platform of Paraíba State. *R* and *p* values correspond to the similarity analysis performed. (b) Effect of depth on the density of sponges (i.e. number of individual sponges per square meter in each location). Line and band show the prediction and 95% confidence intervals of a Gamma GLMM, while dots show raw data points. The dotted vertical line indicates the 30 m depth threshold, which is considered the division between shallow and deep reefs. m*R* ^2^ = marginal *R* ^2^, c*R* ^2^ = conditional *R* ^2^, and *p* = *p*‐value indicating the significance of the relationship.

We observed a marked variation in the composition of sponge species across the depth gradient (Figure 3a). Notably, more than 40% of the variation we observed was significantly correlated with the difference in depth, according to our similarity analysis. This suggests that depth plays a substantial role in determining the makeup of sponge communities, with distinct assemblages preferring specific depth ranges. Furthermore, our study revealed a notable increase in sponge density with depth. Specifically, we found that deep locations exhibited an average sponge density (ind/m^2^) approximately eight times greater than that of shallow reef locations (Table 1). This relationship between depth and sponge density was statistically supported by our model, which demonstrated a significant positive effect of the depth on the density of sponges (i.e. number of individual sponges per square meter in each location) across the investigated depth gradient (Figure 3b).

**FIGURE 4:**
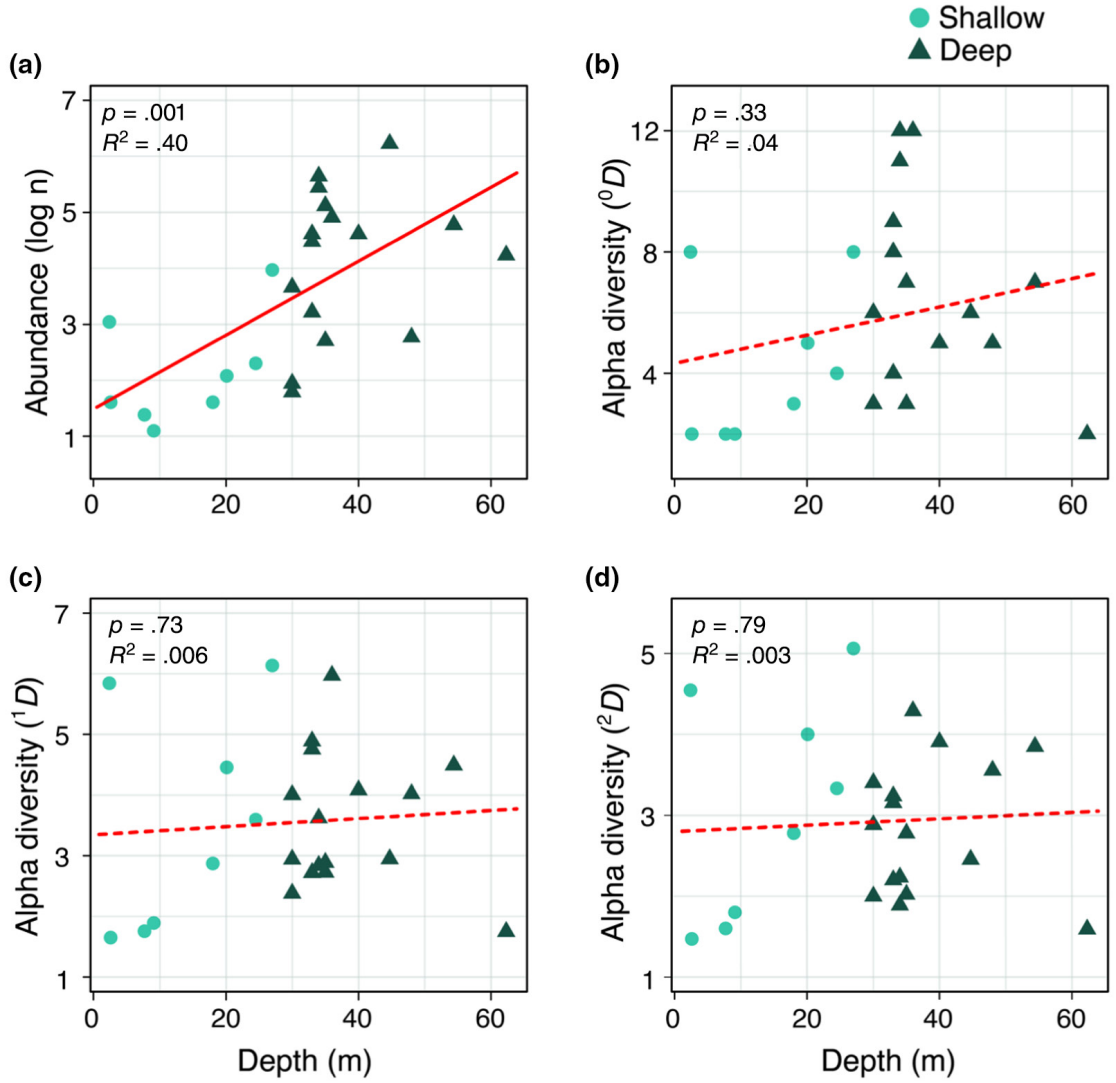
Abundance and alpha diversity for rare (^0^ *D*_α_), typical (^1^ *D*_α_) and dominant (^2^ *D*_α_) species along a depth gradient in the continental platform of Paraíba State. Values shown in panel (a) represent the number of individuals per site (i.e., general abundance), while values shown in panels (b–d) represent the effective number of species recorded in the 24 sampled locations. Solid red line represents a significant statistical interaction, while dashed red lines represent a non‐significant interaction.

Our data also revealed a significant positive correlation between the depth of the habitat and the general abundance of sponges (Figure 4a). Conversely, alpha diversity for rare (^0^ *D*_α_), typical (^1^ *D*_α_), and dominant (^2^ *D*_α_) species, respectively, varied from 1 to 12 species per reef location, but were not significantly correlated with depth (Figure 4b–d).

**FIGURE 5:**
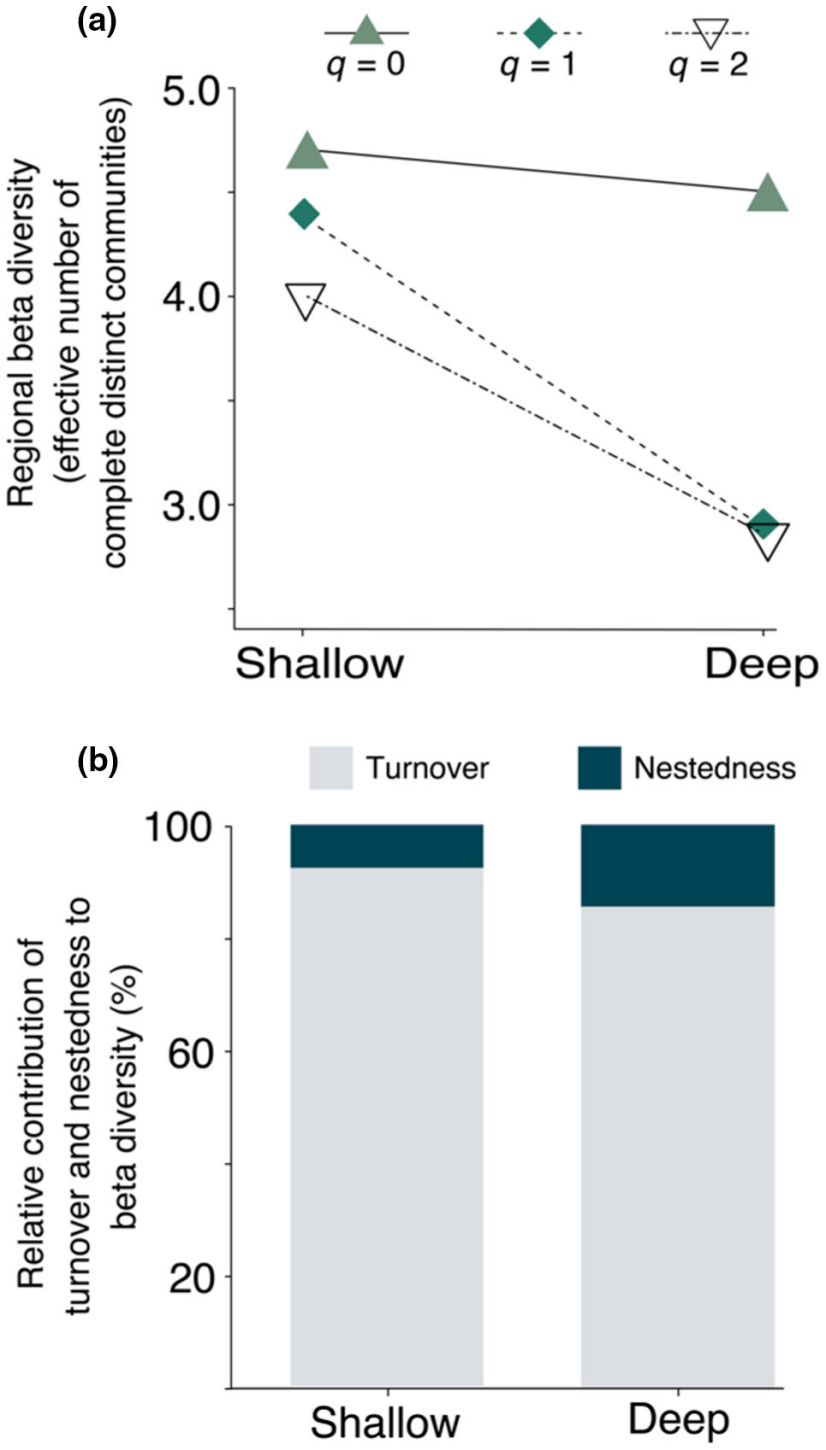
(a) Comparison of beta diversity of rare (^0^ *D*_β_), typical (^1^ *D*_β_) and dominant (^2^ *D*_β_) species between depth categories in the continental shelf of Paraíba State. Values are expressed as the number of completely distinct communities for eight shallow reefs and for 16 deep reefs. (b) Relative contribution of turnover and nestedness contribution of beta diversity between depth categories.

Regarding beta diversity, which assesses the variation in species composition between habitats, our findings aligned with the anticipation of higher indices in shallow reefs. Specifically, beta diversity of rare species was virtually the same between shallow and deep habitats (^0^ *D*_β shallow_ = 4.7 vs. ^0^ *D*_β deep_ = 4.5), while beta diversity of typical and dominant species was higher in the shallow areas (^1^ *D*_β shallow_ = 4.4 vs. ^1^ *D*_β deep_ = 2.9; ^2^ *D*_β shallow_ = 4.0 vs. ^2^ *D*_β deep_ = 2.9) (Figure 5a).

Regarding the relative contribution of turnover and nestedness to beta diversity, turnover was the main driver accounting for 92.7% and 95.7% of the beta diversity of deep and shallow regions, respectively (Figure 3b). Moreover, when we collapsed all shallow and deep communities into two communities and performed the shallow vs. deep comparison, 96.4% of the beta diversity was attributed to turnover.

## 4 DISCUSSION

As global warming continues unabated, coral bleaching events are expected to be increasingly frequent, long‐lasting, and severe (Trisos et al., 2020; Vercelloni et al., 2020). With these events causing a substantial loss in coral diversity and abundance (Morais et al., 2021; Pereira, Lima, Pontes, et al., 2022; Sully et al., 2019), other benthic groups, such as soft corals and sponges, may become essential to delivery functions previously performed by hard corals (Bell, 2008; Bell et al., 2020; Coppock et al., 2022). However, it is still not clear how sponge diversity and abundance and density change along the depth gradient (Lesser, 2006; Lesser & Slattery, 2019), mainly in marginal reef habitats such as the Southern Atlantic. Here, beyond identifying the sponge's community on the shallow and deep reefs, our findings concur with previous studies that reported an increase of sponge abundance and density with depth (Lesser, 2006; Lesser & Slattery, 2019). Conversely, our results about diversity showed that the effective number of rare (^0^ *D*_α_), typical (^1^ *D*_α_) and dominant (^2^ *D*_α_) species did not vary with depth categories, but shallow areas presented greater beta diversity of typical (^1^ *D*_β_) and dominant (^2^ *D*_β_) species than deep areas, as expected based on the greater environmental heterogeneity at smaller depths. Our analyses also demonstrated that turnover is the main driver of beta diversity at any depth, suggesting that mass effects are less important than species sorting in structuring the sponge metacommunity between 2 and 62 m depth. Overall, our findings indicate that this depth interval has a significant influence on sponge abundance, density, taxonomic composition and beta diversity, but not on alpha diversity.

The well‐established understanding is that marine biota, especially benthic groups, typically exhibit changes in abundance, density, and biomass with varying depths (Duckworth & Wolff, 2007; Medeiros et al., 2021; Semmler et al., 2017). However, when talking about sponges, it is also assumed that there is an increase in diversity with depth (Lesser, 2006; Lesser et al., 2020), and this increase would be linked to food availability for filter‐feeding organisms, allowing more individuals of different species to get established in the area (Lesser & Slattery, 2013). Indeed, the high primary production associated with great depths makes more food resources available for sponges (i.e. carbon and nitrogen) when compared to shallower areas (Lesser & Slattery, 2020). Sponges take advantage of the particulate and dissolved organic matter, especially those from picoplankton (Ribes et al., 2003), linking benthic and pelagic communities (Diaz & Rützler, 2001; Lesser & Slattery, 2013; Witman et al., 2004). Such linkage is also closely related to nutrient cycling in coral reefs (de Goeij et al., 2013). However, while this process may have had a clear effect on sponge abundance and density (ind/m^2^) in our study locations, it does not appear to impact sponge alpha diversity. Nevertheless, it is important to note that in our random transect distribution, there are twice as many locations in deep areas as in shallow ones (see Figure 1), which could suggest that the higher abundance found in deep areas is a result of the larger sampling area. However, upon accounting for the surveyed area and determining the sponge density as individuals per square meter (ind/m^2^), it was observed that the average density in deeper zones exceeded that of shallow regions by more than eightfold. Furthermore, our analysis revealed that depth has a substantial and statistically significant impact on sponge density. Likewise, in our analyses, we compared sponge abundance on a per‐location basis rather than using average across different depth categories. Therefore, despite the difference in the number of sampling locations, the abundance and density in deep areas are consistently higher. It is also important to note that our results are based on areas with up to 60 m depth, which, in our case, are near the continental break (~75 m; Morais and Santos (2018)). Future research may shed light on patterns at greater depths, considering that sponges are also present in aphotic zones (Garcia‐sais, 2010). Understanding whether these deep‐sea sponges exhibit similar dynamics as seen here will contribute significantly to our knowledge of marine biodiversity and ecosystem dynamics.

Marine biota responds to depth by generating different spatial alpha and beta patterns (e.g. Medeiros et al., 2021; Morais & Santos, 2018). In our study, species composition changes between shallow and deep areas, although one‐third (13 of 36 spp.) of species occurred in both depth categories. This generates an expressive number of completely distinct communities across the continental shelf. Notably, this diversity is even more pronounced in the shallower areas, as also observed for corals in the same study area (Morais & Santos, 2018). On the other hand, in a local (alpha) scale, depth seems to have a weak effect on sponge diversity (Figure 4b–d) with the effective number of species varying substantially within the same depth range. For example, within a specific depth range of 30–35 m, there is a notable variation in diversity across different reefs; some are characterized by a species richness of only 3, while others boast as many as 12 distinct species (^0^ *D*_α_, Figure 4b). This inconsistency suggests that microhabitat diversity, encompassing various ecological niches and growth forms, plays a pivotal role in determining local species distribution. In each of these microhabitats, areas with different adverse, but not impeditive conditions, can be found (Wulff, 2012). For example, substrates with contrasting characteristics may be determinants of larval setting (Whalan et al., 2008), intra and interspecific competition (González‐Murcia et al., 2023; Liddell & Avery, 2000), predator presence or absence, hydrodynamical conditions (Hill, 1998; Pawlik et al., 2015; Wulff, 2005), and sedimentation rates (Tjensvoll et al., 2013). Therefore, these characteristics could account for the observed disparities in species richness among locations sharing the same depth. Thus, our findings reveal that depth is a relatively poor predictor of sponge alpha diversity in marine zones reaching down to 60 m, suggesting that a variety of factors other than depth significantly influence the alfa diversity of sponge species in these habitats.

Surprisingly, nestedness had a weak association with beta diversity along the depth gradient. In fact, species turnover was responsible for more than 90% of beta diversity between reefs, not only within shallow and deep regions but also when comparing shallow and deep (Figure 5). Contrary to initial expectations, the relative contribution of species turnover was also high in deep areas. The low contribution of nestedness indicates the lack of a big, hyper‐diverse, local community that exports a subset of its species to a less diverse counterpart (*mass effects* sensu Leibold et al., 2004) (Figure 5b). Indeed, the high contribution of species turnover suggests that a considerable number of species are localized within a limited number of communities, likely in pursuit of the most favorable biotic and/or abiotic conditions for their survival, growth, and reproduction (Abdul Wahab et al., 2014; Leibold et al., 2004). As also observed in this area for corals and reef fishes (Medeiros et al., 2021; Morais & Santos, 2018), species turnover is the rule at any depth up to 60 m, even at depths where beta diversity is relatively low. Therefore, it remains imperative to protect sponge communities at all depths in order to preserve and maintain the overall regional (gamma) diversity.

It is important to note that the findings presented herein have primarily focused on the depth gradient as a singular variable impacting marine sponge communities. However, a multitude of other environmental and physical factors that do not necessarily vary linearly with depth may also play a critical role in shaping these communities. Among these, temperature, light intensity, and hydrostatic pressure stand out as pivotal elements that could significantly influence sponge distribution and growth (Hinderstein et al., 2010). For instance, temperature, salinity, depth, and nutrients/oxygen together may explain around 25% of microbiome variations in sponges, potentially impacting their growth rates and reproductive success (Busch et al., 2022). Similarly, light intensity directly influences photosymbiotic relationships that certain sponge species maintain, which are crucial for their energy acquisition and survival, especially in the euphotic zone (Lemloh et al., 2009; Pineda et al., 2016). While our study has not directly investigated these factors, their significance cannot be understated, and they warrant further exploration to fully understand the complex interplay driving sponge diversity and abundance in marine ecosystems.

Our results also emphasize understudied aspects regarding the biological diversity of marginal reefs (Soares et al., 2021). In this study, we demonstrate how sponges in marginal reef formations have their diversity vertically and horizontally distributed in Northeast Brazil. The ecological implications of this finding deserve further investigation as highlighted in other studies. For example, distinct organisms occurring in the marginal reef system at the Amazon River mouth have their distribution limited by its characteristic environmental features (e.g. high sedimentation rates, strong winds, and currents caused by river discharge) (Francini‐Filho et al., 2018; Moura et al., 2016). Under this perspective, sponges can colonize these suboptimal limiting areas in which there is a low species diversity of other biological benthic groups (Moura et al., 2016). Similarly, marginal reefs of the Southwestern Atlantic, specifically those found in the Northeast region (this study) are also subjected to suboptimal conditions (Soares et al., 2018) and can also offer considerable available space for such resistant marine organisms. However, even resistant organisms like sponges are subject to environmental adversities caused by human activities, such as pollution (Zaneveld et al., 2016) and mining (Fettweis et al., 2010), or even on a bigger scale, such as ocean warming (Lesser & Slattery, 2020; Tittensor et al., 2010). Consequently, it is vital to include marginal reefs as a priority in the international agenda for research and the conservation of marine ecosystems due to their unique futures and their capacity to sustain unique biological organisms (Soares et al., 2021; Sommer, 2022).

Overall, our results elucidated the depth‐related dynamics of sponge communities in marginal reef ecosystems, revealing that while sponge abundance and density significantly increase with greater depth, alpha diversity remains consistent across the depth gradient. Beta diversity is higher in shallow waters, driven by environmental heterogeneity rather than depth. Our findings also highlight turnover as the primary driver of species variation. It emphasizes that shallow and deep areas complement each other, and conservation efforts must encompass the entire depth range to protect the metacommunity structure and maintain regional biodiversity.

## AUTHOR CONTRIBUTIONS

**Juliano Morais:** Conceptualization (equal); data curation (equal); formal analysis (equal); investigation (equal); writing – original draft (lead); writing – review and editing (equal). **Igor L. Cordeiro:** Data curation (equal); formal analysis (supporting); investigation (equal); writing – review and editing (supporting). **Aline P. M. Medeiros:** Conceptualization (equal); data curation (equal); formal analysis (equal); investigation (equal); writing – original draft (equal); writing – review and editing (equal). **George G. Santos:** Conceptualization (equal); investigation (equal); methodology (equal); supervision (supporting); writing – original draft (equal); writing – review and editing (equal). **Bráulio A. Santos:** Conceptualization (equal); data curation (equal); funding acquisition (lead); investigation (equal); supervision (lead); writing – original draft (equal); writing – review and editing (equal).

## FUNDING INFORMATION

This study was funded by Fundação Grupo Boticário de Proteção à Natureza (1044–20152). The Conselho Nacional de Desenvolvimento Científico e Tecnológico (CNPq) provided a graduate scholarship to ILC and a research productivity fellowship to BAS (312178/2019–0). The Programa Institucional de Internacionalização (PrInt) of CAPES (Brazilian Federal Agency for Support and Evaluation of Graduate Education, Ministry of Education of Brazil), Fundação de Apoio à Pesquisa do Estado da Paraíba (Fapesq‐PB), and the PADI Foundation (application #32777) provided a scholarship to APMM. GGS also received support from CNPq (Edital no. 28/2018—Universal 432900/2018–7). BAS acknowledges UFPB for the scientific production support (PVA‐13357‐2020).

## Data Availability

The data that support the findings of this study are openly available in the Zenodo repository at https://doi.org/10.5281/zenodo.10867112.
